# A robust family of Golden Gate *Agrobacterium* vectors for plant synthetic biology

**DOI:** 10.3389/fpls.2013.00339

**Published:** 2013-09-02

**Authors:** Shahram Emami, Muh-ching Yee, José R. Dinneny

**Affiliations:** Department of Plant Biology, Carnegie Institution for ScienceStanford, CA, USA

**Keywords:** molecular cloning, *Agrobacterium* binary vectors, synthetic biology, combinatorial libraries, Golden Gate Cloning, plant transformation

## Abstract

Tools that allow for rapid, accurate and inexpensive assembly of multi-component combinatorial libraries of DNA for transformation into plants will accelerate the progress of synthetic biology research. Recent innovations in molecular cloning methods has vastly expanded the repertoire with which plant biologists can engineer a transgene. Here we describe a new set of binary vectors for use in *Agrobacterium*-mediated plant transformation that utilizes the Golden-Gate Cloning approach. Our optimized protocol facilitates the rapid and inexpensive generation of multi-component transgenes for later introduction into plants.

## Introduction

Assembly and transformation of a multi-component DNA construct such as a promoter and a reporter gene fusion is a common task in everyday plant research. Therefore optimization of this process is likely to yield major productivity gains. Traditionally, restriction endonuclease-mediated cleavage in combination with T4 DNA ligase-mediated joining has been used to create the desired DNA construct. However this method is time consuming, sequence dependent and is not well suited for high-throughput assembly of a large number of constructs.

The advent of recombination-based cloning utilizing λ phage Integrase (Hartley et al., 2000), which was later commercialized by Invitrogen as *Gateway*® *Recombination Cloning* reduced the time needed to assemble a construct. The later version of this strategy (*MultiSite Gateway*®) allowed for simultaneous cloning of up to three DNA fragments into a vector by utilizing mutant versions of the λ phage Integrase recognition sequences that are only functional in specific combinations (Petersen and Stowers, 2011). However, Gateway cloning has significant drawbacks, which include the high cost of cloning kits, the need to use specific bacterial strains to propagate plasmids carrying the *ccdB* gene and the presence of recombination scars in the final product that can have effects on gene expression or protein function.

Other non-Integrase based cloning techniques have been used in order to replace the traditional restriction endonuclease/ligase methods. Many of these techniques use the homology between different fragments of partially single-stranded DNA to anneal the fragments together and build the desired construct. They differ mainly in how the partially single-stranded DNA is initially generated. For example in Uracil Specific Excision Reagent (USER) cloning (Nour-Eldin et al., 2010), uracil bases are first incorporated into each DNA fragment using primers containing uracil instead of thymine. Uracil DNA Glycosylase (UDG) enzyme is then used to excise the uracil bases. Subsequent treatment of the excision site with DNA glycosylase-lyase Endonuclease VIII enzyme will result in a partially single-stranded DNA fragment.

Sequence and Ligase Independent Cloning (SLIC; Li and Elledge, 2012) uses the 3′ exonuclease activity of the T4 DNA polymerase, which is heavily favored in the absence of dNTPs in order to create partially single-stranded DNA fragments. Addition of the dCTPs will force the T4 DNA polymerase to switch from exonuclease to polymerase activity, and the absence of dATPs, dGTPs, and dTTPs results in a paused polymerase. Once different partially single-stranded fragments have been mixed and annealed to their targets, they will be transformed into *E. coli*, where the nicks are repaired.

Gibson cloning (Gibson, 2011) uses T5 exonuclease, which removes nucleotides in the 5′–3′ direction to create a DNA fragment with a 3′ single-stranded overhang. As in USER and SLIC, fragments are annealed together based on sequence homology at these single-stranded ends. Phusion DNA polymerase is used to fill in the gaps between the annealed DNA fragments and ligase seals the nicks. In general, homologous regions are recommended to be ~25 base pairs (bps) in length although in many cases ≥15 bps overlap can work. In our experience, 70–80% of the colonies generated via Gibson cloning of two DNA fragments, have the desired construct (data not shown).

USER, SLIC and Gibson cloning methods enable the joining of DNA fragments without intervening unwanted DNA sequences. Gibson has been demonstrated to work well for ligating relatively large DNA fragments and was used to assemble the genome of *Mycoplasma mycoides* (Gibson et al., 2010). Both SLIC and Gibson cloning also have a number of weaknesses: (1) repeats in the homologous regions used to anneal DNA fragments can result in undesired side products (2) single-stranded DNA that has a stable secondary structure such as a hair-pin, will not base-pair with its target (3) fragments smaller than 250 bps could be completely digested by the exonuclease before annealing, so optimization may be needed when working with fragments of this size class.

In Circular Polymerase Extension Cloning (CPEC; Quan and Tian, 2011) linear insert(s) and destination vector are first heat denatured, creating single-stranded DNA fragments that can anneal to their targets using their overlapping sequences. Subsequent DNA polymerase extension allows the previously single-stranded DNA fragments to act as primers to regenerate the desired DNA sequences as insert(s) in the destination vector. The overlapping sequences between inserts and the destination vector need to be carefully designed to be unique and have very similar (±2°C) high melting temperatures (*T*_*m*_ ~ 60–70°C) to minimize nonspecific-hybridization. Due to the dependence of this technique on DNA polymerase extension there is likely to be an upper bound in terms of the size of the final assembly, although an 8.4 kb plasmid assembled from four fragments has been previously reported (Quan and Tian, 2009).

While in theory it is possible to generate combinatorial libraries of constructs, using any of the methods mentioned above, in practice the demonstrated examples have been few and far between. However high-throughput combinatorial libraries of synthetic constructs have been implemented with ease using the Golden Gate Cloning strategy (Cermak et al., 2011; Engler and Marillonnet, 2011).

**Figure 1:**
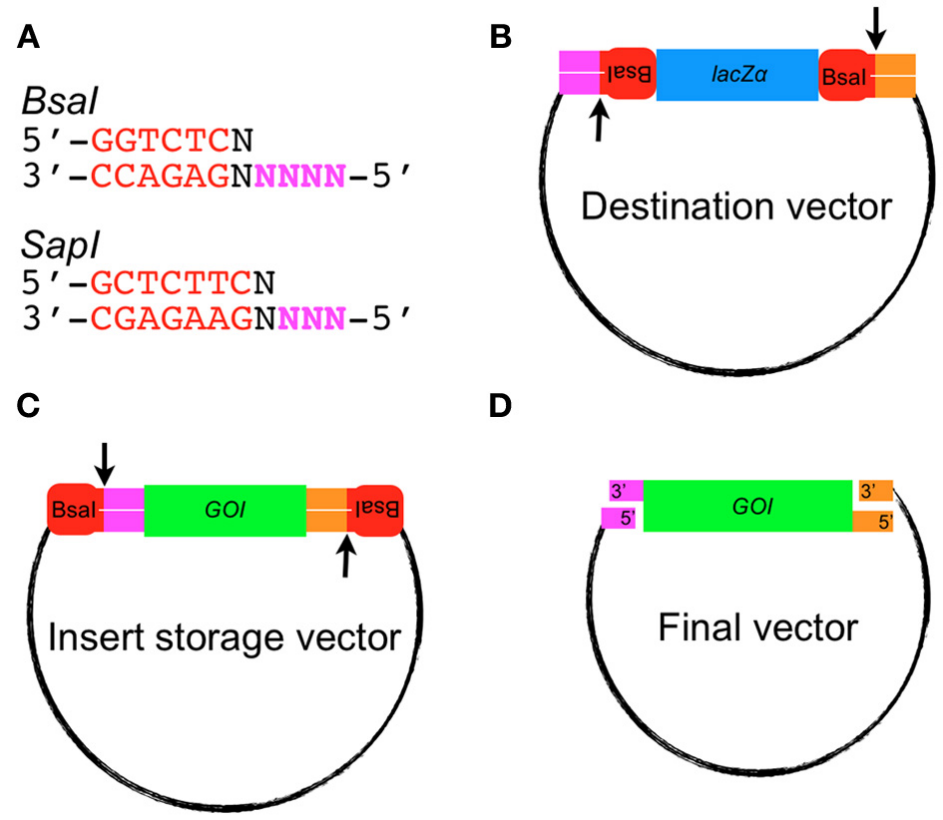
**Golden Gate assembly of an insert into the destination vector. (A)** Recognition sequences for the Type IIS restriction endonucleases *BsaI* and *SapI*. 5′ overhang sequences used for annealing fragments are shown in bold magenta lettering. **(B)** Example of a Golden Gate-compatible destination vector. Note the orientation of the *BsaI* sites cause excision of the *lacZ*α gene. **(C)** Example of a Golden Gate compatible vector containing a gene of interest (GOI) that will be released after digestion with *BsaI*. **(D)** A typical Golden Gate Cloning reaction would involve mixing the destination vector and insert storage vector together into one tube at equal molar ratio with *BsaI* and T4 DNA ligase. The final vector produced would lack *BsaI* recognition sequences and be resistant to digestion.

Golden Gate Cloning (Engler et al., 2008, 2009) uses Type IIS restriction enzymes. These enzymes cut at a single site outside of their recognition-binding site sequence. For example *BsaI* recognizes the sequence 5′-GGTCTC-3′, cleaves DNA one bp 3′ of the recognition site, and creates a 5′ overhang that is four bases in length. The 5′ overhang sequences can be designed to allow for annealing different fragments together. In a typical Golden Gate Cloning reaction, each fragment to be assembled into the destination vector is flanked by *BsaI* sites that, when cleaved, generate unique overhang sequences on either side of the insert. The destination vector will typically have *BsaI* sites that linearize the plasmid when digested and generate overhang sequences for ligation of the insert(s) (Figures 1, A1). The fragments are concatenated together using DNA ligase to generate the desired product. The digestion and ligation reactions take place in the same tube using alternating temperatures to drive the ligation and digestion reactions. Assembly of the correct vector is processive since ligation of the insert with the vector does not recreate the *BsaI* recognition sequence.

Here we describe a set of binary vectors for *Agrobacterium*-mediated plant transformation based on the Golden Gate Cloning strategy that can be utilized to generate transgenes of variable size and composition. We have also developed a binary vector that uses *SapI*, a Type IIS enzyme that uses a 7-bp recognition sequence and allows for additional flexibility when performing Golden Gate Cloning.

## Results

**Figure 2:**
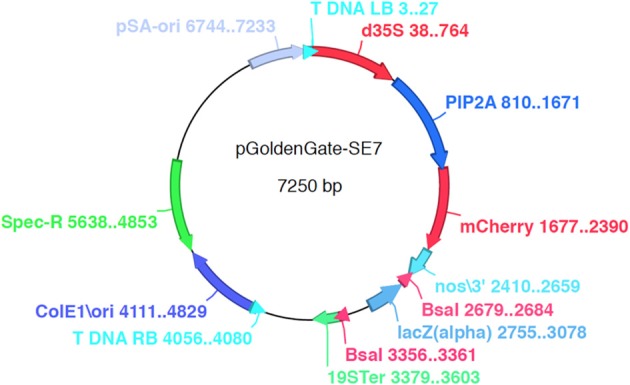
**pGoldenGate-SE7 map**.

Three *Agrobacterium* binary destination vectors (pGoldenGate-SE7, pGoldenGate-SE9, pGoldenGate-MCY2) (Figures 2, A2, A3) have been constructed. All three plasmids use Spectinomycin resistance as a selectable marker in *E. coli* and *Agrobacterium*.

**Figure 3:**
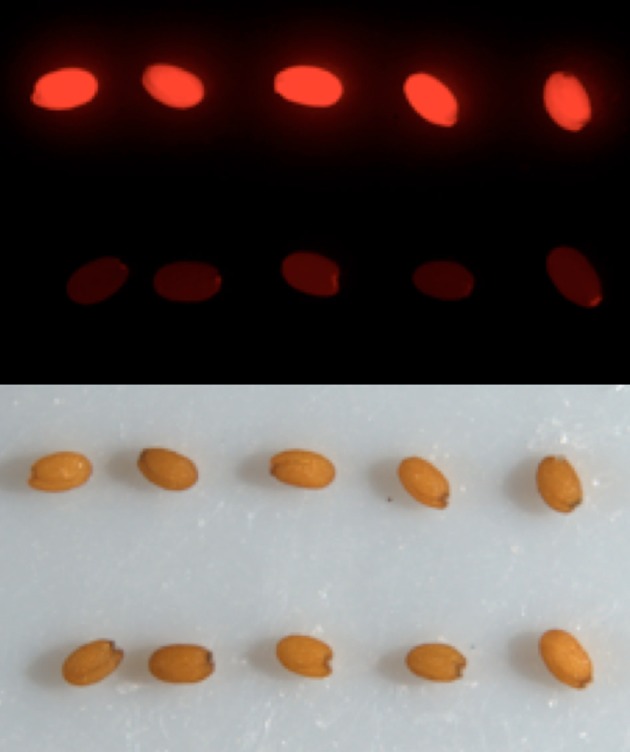
***Pro35S:PM-mCherry* cassette (pGoldenGate-SE7, pGoldenGate-MCY2) enables the selection of transgenic (top row) vs. non-transgenic (bottom row) seeds**. Fluorescence (top image) and bright field (bottom) images are shown.

pGoldenGate-SE7 and pGoldenGate-MCY2 both use the *Pro35S:PM-mCherry* transgene as the selectable marker in plants. *Pro35S:PM-mCherry* drives expression of a plasma-membrane-localized mCherry constitutively in the plant including in the mature seeds (Figure 3). This selectable marker allows for the selection of *T*_1_ transgenic plants by screening for red fluorescence in the dry seeds. Thus, seedlings are not exposed to antibiotics or herbicides that may have a negative effect on normal growth and development, and *T*_1_ plants can be used for analysis. The *Pro35S:PM-mCherry* reporter is also useful at later stages of transgenic line characterization for scoring the segregation ratio of the transgene. Use of this selection method is compatible with high-throughput selection approaches such as automated seed sorting (http://www.unionbio.com/copas). We have also generated the pGoldenGate-SE9, which uses Kanamycin resistance as an alternative selection marker in plants.

Our family of vectors include the *lacZ*α gene cloned between two *BsaI* or *SapI* sites, which allows for selection of recombinant clones based on blue-white colony color. The *lacZ*α gene is flanked by *BsaI* sites on both sides of the selection marker, and is excised from the vector upon digestion with *BsaI*. The excision of *lacZ*α will result in two four-base overhangs on opposite strands shown here in lowercase letters (3′-GAGCTC*tcat*-5′) and (5′-*tgga*AAGCTT-3′) adjacent to the underlined *XhoI* and *HindIII* sites on the destination vectors (pGoldenGate-SE7, pGoldenGate-SE9).

pGoldenGate-MCY2 uses *SapI* instead of *BsaI* as the Type IIS enzyme. *SapI* has a seven bp recognition sequence (5′-GCTCTTC-3′), and thus occurs less frequently in genomes than *BsaI*. *SapI* cleaves DNA one bp 3′ of the recognition site, and creates a 5′ overhang that is three bases in length. The three base overhangs allows for scar-less assembly of coding DNA sequences, since each codon is also three bps long. Digestion of pGoldenGate-MCY2 with *SapI* will result in two three-base overhangs on opposite strands shown here in lowercase letters (3′-GAGCTC*tca*-5′) and (5′-*tgg*AAGCTT-3′) adjacent to underlined *XhoI* and *HindIII* sites in the destination vector.

**Figure A1:**
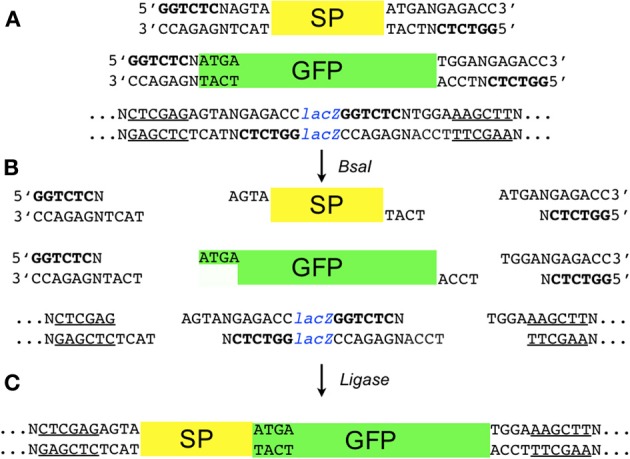
**Detail schematic of the two-component assembly using pGoldenGate-SE7 as the destination vector**. **(A)** Synthetic promoter (SP), reporter (GFP), and the pGoldenGate-SE7 destination plasmid containing the *lacZ*α gene are put in the same reaction tube along with *BsaI* and the T4 DNA ligase. The recognition site for *BsaI* (5′-GGTCTC-3′) is shown in bold. The underlined segments are *XhoI* and *HindIII* sites flanking the *lacZ*α gene in the destination vector. **(B)** Shows the partially single-stranded DNA fragment generated after the digestion with *BsaI* **(C)** Ligase is used to assemble the desired product. Note that 5′-ATGA-3′ is the first four bps of the GFP coding DNA sequence and therefore this assembly is scar-less.

**Figure A2:**
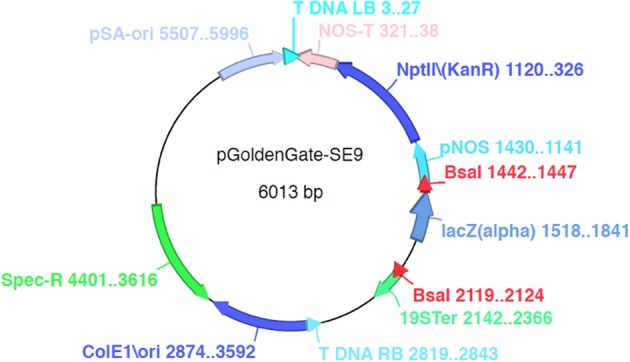
**pGoldenGate-SE9 map**.

**Figure A3:**
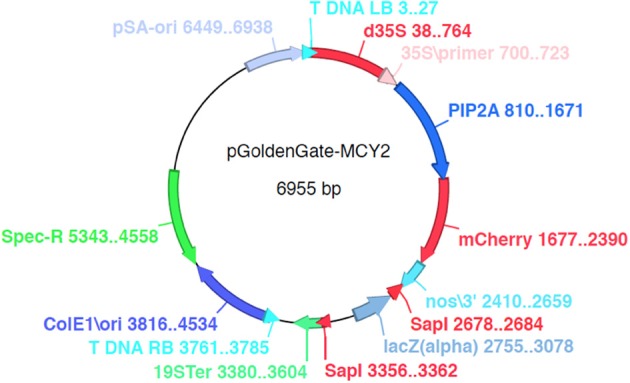
**pGoldenGate-MCY2 map**.

To introduce a transgene into our Golden-Gate vectors, individual components such as promoter and reporter genes are first amplified with PCR primers that incorporate *BsaI* or *SapI* sites with the desired overhangs at each end (Figure A1). These PCR products are cloned for sequence confirmation before use, for example, using pCR™-BluntII-TOPO® (Life Technologies) or other similar PCR-product cloning kits. The pCR-BluntII-TOPO vector itself contains a *BsaI* site, so it's important to avoid using its overhang sequence (5′-*gtta*-3′) when designing *BsaI* Golden Gate overhangs. The pCR-BluntII-TOPO vector also contains two *SapI* sites with overhangs (5′-*tcc*-3′) and (5′-*gca*-3′) which should be avoided when designing *SapI* Golden Gate overhangs.

To maximize success, all plasmids are first quantitated (e.g., by Qubit, Life Technologies) and then combined in an equal molar ratio in the reaction as shown in Table 1. We have been able to easily assemble five fragments in a single reaction using the pGoldenGate-SE7 vector. A detailed schematic of the two-component assembly using pGoldenGate-SE7 as the destination vector is shown in Figure A1. The largest size of a given insert fragment tested thus far was 3500 bps, and the smallest fragment was only three bps between the two single-stranded overhangs. On average we obtain about three blue colonies for every 1000 white colonies for the Golden Gate reactions. Remarkably of the 46 cloning reactions performed with pGoldenGate-SE7, the success rate of obtaining the correct multi-component plasmid was 100% even when only one white colony was screened per construct. We have successfully assembled constructs using inserts that were either first cloned into plasmids, generated by annealing pairs of short-oligonucleotides or as gel-purified PCR products.

**Table 1:** **Assembly of Golden Gate reaction**.

**Components**	**Amount**
Destination vector (e.g. pGoldenGate-SE7)	100 ng
Each additional assembly piece	In equal molar concentration to destination vector
10X NEB T4 DNA ligase buffer	1.5 μl
10 mM ATP	1.5 μl
*BsaI* or high concentrated *SapI*	1.0 μl
NEB T4 DNA ligase (2 million cohesive end units/ml)	1.0 μl
dH_2_O	To bring the total volume to 15 μl

We have used the Golden Gate assembly protocol from J5 (http://j5.jbei.org/j5manual/pages/81.html), except that supplementary ATP is added to optimize ligase activity. Single use aliquots of 10X Ligase buffer and 10 mM ATP are made to reduce freeze-thaw cycles of those buffers. In our experience the addition of more than 1 μl of T4 DNA ligase or 0.75 μl of high concentrated T4 DNA ligase can actually reduce the efficiency of the Golden Gate Cloning reaction. For *SapI*, it is important to use the high concentration stock (10,000 units/ml) of *SapI* enzyme. The PCR cycling parameters are shown in Tables 2, 3. In our experience 25 repetitions of the first cycle is more that adequate to assemble two fragments of DNA (Table 2). We have used up to 50 repetitions of the first cycle in order to successfully assemble up to five fragments of DNA. 80°C heat treatment is used to inactivate both the *BsaI* and DNA ligase. Before transformation into *E. coli*, 1 μl of fresh *BsaI* is added to the reaction mix (for pGoldenGate-SE7 and pGoldenGate-SE9 plasmids only). The addition of *BsaI* after the inactivation of DNA ligase reduces the number of blue colonies to virtually zero. The total reaction volume before the addition of fresh *BsaI* is 15 μl. For *SapI*, no additional digestion is needed after the 25 cycles of digestion and ligation.

**Table 2:** **PCR cycling parameters for Golden Gate Cloning reaction where there are no internal *BsaI/SapI* sites within the insert fragment(s)**.

37°C 2 min.	25–50X
16°C 5 min.	
80°C 10 min.	
^‡^37°C 30 min.	

‡ Before the start of this step, 1 μl of fresh BsaI needs to be added to the reaction mix.

**Table 3:** **PCR cycling parameters for Golden Gate Cloning reaction where *BsaI* site(s) exist within the insert fragment(s)**.

37°C 2 min.	25–50X
16°C 5 min.	
80°C 10 min.	
^*^16°C 2 h	

* Before the start of this step, 1 μl of fresh DNA ligase needs to be added to the reaction mix.

Destination vector (pGoldenGate-SE7 or pGoldenGate-SE9) is first digested with BsaI, and the non-lacZα fragment of the digestion is gel-purify. The non-lacZα fragment is used in place of the destination vector in the Golden Gate Cloning reaction with the PCR cycling parameter as shown.

Additional *BsaI* site(s) within one or more of the DNA fragments being used can complicate cloning, since digestion at such sites (1) might generate overhang sequences that interfere with ligation of the intended construct, and (2) will cause cleavage of the vector prior to transformation into *E. coli*. To circumvent this problem, we first treat the destination vector with *BsaI*, and gel-purify the non-*lacZ*α fragment of the digestion. The non-*lacZ*α fragment will be used in place of the destination vector in the Golden Gate Cloning reaction with the PCR cycling parameter as shown in Table 3. In order to eliminate the possibility of nonspecific-hybridization to any partially single-stranded DNA other than its intended target, we design each overhang so that it differs in sequence from its non-target overhangs by at least two bps.

## Conclusion

Until fairly recently, assembling a multi-component DNA construct was considered a project bottleneck. Thanks to what seems to be an ever-expanding arsenal of molecular tools and techniques, rapid, accurate and inexpensive assembly of multi-component constructs is now possible. The decreasing cost of gene synthesis allows for the construction of combinatorial multi-component DNA libraries where one or more parts may have variable sequences. Golden Gate Cloning allows for these multi-component libraries to be easily assembled and the use of fluorescence-based selection markers enable high-throughput methods for selection of transgenic plants.

## Materials and methods

Invitrogen's One Shot® TOP10 chemically competent *E. coli* cell strain, which is *lacZ*Δ*M15*, was used to allow for blue/white color screening of colonies. Cells were grown on LB/Agar plates with 40 μg/ml each of Spectinomycin and X-gal. *BsaI (R0535L)*, *SapI* (R0569M, 10,000 units/ml), T4 DNA ligase (M0202M) were obtained from New England Biolabs. DNA quantitation was done using the Qubit dsDNA HS Assay kit (Life Technologies Q32854). Gel purification of plasmid DNA was done on agarose gels stained with Crystal Violet (C3886 Sigma) 10 ug/ml. Gels were visualized in room light to avoid damage from UV-trans-illuminators. Excised gel fragments were purified using Qiagen's Qiaquick Gel Extraction Kit. Table 1 shows the reaction mix used in a typical Golden Gate assembly and Tables 2, 3 shows the PCR cycling parameters. Table 4 shows the sequences needed to add the *BsaI* or *SapI* sites to the PCR products prior to Golden Gate Cloning for a two-part (promoter plus reporter) scar-less assembly into the destination vector. Plasmid sequences and ordering information can be obtained through Addgene.org using the following identification numbers: (pGoldenGate-SE7: 47676), (pGoldenGate-SE9: 47677), (pGoldenGate-MCY2: 47679).

**Table 4:** **Sequences to add *BsaI* or *SapI* sites to the PCR products prior to Golden Gate Cloning for a two-part (promoter plus reporter) scar-less assembly into the destination vector**.

**Primer**	**Recognition/Overhang**	**Sequence to add**
*BsaI* forward promoter	5′-GGTCTCN**AGTA**-	Starting from the first bp of your promoter
*BsaI* reverse promoter	5′-GGTCTCN**TCAT**- ^1^	Reverse complement of your promoter starting from the last bp
*BsaI* forward reporter	5′-GGTCTCN**ATGA**- ^1^	Starting from the 5th bp of your reporter
*BsaI* reverse reporter	5′-GGTCTCN**TCCA**-	Reverse complement of your reporter starting from the last bp
*SapI* forward promoter	5′-GCTCTTCN**AGT**-	Starting from the first bp of your promoter
*SapI* reverse promoter	5′-GCTCTTCN**CAT-** ^2^	Reverse complement of your promoter starting from the last bp
*SapI* forward reporter	5′-GCTCTTCN**ATG-** ^2^	Starting from the 4th bp of your reporter
*SapI* reverse reporter	5′-GCTCTTCN**CCA**-	Reverse complement of your reporter starting from the last bp

1 Assumes the first 4 bps of your reporter is 5′-ATGA-3′.

2 Assumes the first 3 bps of your reporter is 5′-ATG-3′.

BsaI primers apply to the pGoldenGate-SE7 and pGoldenGate-SE9 plasmids, whereas SapI primers apply to the pGoldenGate-MCY2 plasmid. Overhangs are shown in bold.

## Plasmid construction

### pGoldenGate-SE7

The pCherry-pickerT plasmid (Duan et al., 2013) was used as a starting point for construction of our Golden Gate vectors. An existing *BsaI* site located outside of the *ccdB* gene was first mutated using the QuickChange® Site-Directed Mutagenesis kit (Stratagene catalogue # 200519). A *lacZ*α gene fragment isolated from pUC19 was PCR amplified to include *BsaI* recognition sites and cloned between the *HindIII* and *XhoI* sites of pCherry-pickerT to replace the *ccdB* gene.

### pGoldenGate-SE9

A Kanamycin resistance gene was PCR amplified (~1400 bps) from the dpGreenKanT plasmid and used to replace the *Pro35S:PM-mCherry* gene in pGoldenGate-SE7 between the *XhoI* and *AscI* restriction enzyme sites to create the pGoldenGate-SE9 plasmid.

### pGoldenGate-MCY2

The *BsaI* cloning sites flanking the *lacZ*α gene in pGoldenGate-SE7 were replaced with *SapI* sites. The resulting PCR product was digested with *XhoI* and *HindIII* and ligated into pGoldenGate-SE7, resulting in pMCY1. A pre-existing *SapI* site and a second copy of the *lacZ* promoter that was downstream of the 19S terminator and the *HindIII* site were next removed. Gibson assembly was used to create the final pGoldenGate-MCY2 plasmid.

### Conflict of interest statement

The authors declare that the research was conducted in the absence of any commercial or financial relationships that could be construed as a potential conflict of interest.
